# Coronary Intravascular Lithotripsy for Treatment of Severely Calcified Lesions: Long-Term Sex-Specific Outcomes

**DOI:** 10.1016/j.jscai.2023.101069

**Published:** 2023-11-07

**Authors:** Jennifer Frampton, Kathleen E. Kearney, J. Dawn Abbott, Dean J. Kereiakes, Carlo Di Mario, Shigeru Saito, Ecaterina Cristea, Robert F. Riley, Jean Fajadet, Richard A. Shlofmitz, Ziad A. Ali, Andrew J. Klein, Matthew J. Price, Jonathan M. Hill, Gregg W. Stone, Alexandra J. Lansky

**Affiliations:** a Section of Cardiovascular Medicine, Department of Internal Medicine, Yale School of Medicine, New Haven, Connecticut; b University of Washington, Seattle, Washington; c Lifespan Cardiovascular Institute and Warren Alpert Medical School of Brown University, Providence, Rhode Island; d The Christ Hospital and the Lindner Research Center, Cincinnati, Ohio; e Careggi University Hospital, Florence, Italy; f Shonan-Kamakura General Hospital, Kamakura, Kanagawa, Japan; g Overlake Medical Center & Clinics, Bellevue, Washington; h Clinique Pasteur, Toulouse, France; i St. Francis Hospital, Roslyn, New York; j Cardiovascular Research Foundation, New York, New York; k Piedmont Heart Interventional Cardiology, Atlanta, Georgia; l Scripps Clinic, La Jolla, California; m Royal Brompton Hospital, London, United Kingdom; n The Zena and Michael A. Wiener Cardiovascular Institute, Icahn School of Medicine at Mount Sinai, New York, New York

**Keywords:** calcification, coronary artery disease, drug-eluting stent, intravascular lithotripsy, sex

## Abstract

**Background:**

Intravascular lithotripsy (IVL) for calcified lesion preparation prior to drug-eluting stent placement has high procedural success and safety, especially in women, whereas other atheroablative approaches are associated with increased procedural complications. We sought to investigate long-term sex-based outcomes of IVL-facilitated stenting.

**Methods:**

We performed a patient-level pooled analysis of the single-arm Disrupt CAD III and IV studies. Patient baseline, procedural characteristics, and outcomes were examined according to sex at 30 days and 1 year. The primary end point was major adverse cardiac events (a composite of cardiac death, all myocardial infarction, or target vessel revascularization). Target lesion failure was defined as cardiac death, target vessel myocardial infarction, or ischemia-driven target lesion revascularization.

**Results:**

A total of 448 patients, 106 (24%) women, were included. Women were older and less likely to be smokers. Women had smaller reference vessel diameters (2.8 mm vs 3.1 mm), shorter lesion length (23.6 mm vs 27.1 mm), and shorter total calcified length (44.4 mm vs 49.3 mm) compared with men. Post-IVL angiographic outcomes and complications were similar between women and men. At 1 year, major adverse cardiac event rates (12.3% vs 13.2%, *P* = .52) were not different between women and men. There were no differences between women and men (10.4% vs 11.2%; *P* = .43) in target lesion failure at 1 year.

**Conclusions:**

Use of IVL in the treatment of severely calcified lesions is associated with low rates of adverse clinical events and with similar safety and effectiveness in women and men at 1 year.

## Highlights

• Intravascular lithotripsy for calcified lesions has high procedural success and safety.
• Other approaches to treat calcified lesions have higher complications in women.
• We investigated long-term sex-based outcomes of intravascular lithotripsy-facilitated stenting.
• At 1 year, major adverse cardiac event rates were not different between men and women.
• Intravascular lithotripsy is associated with low rates of adverse events with similar safety between sexes.

## Central Illustration

**Figure:**
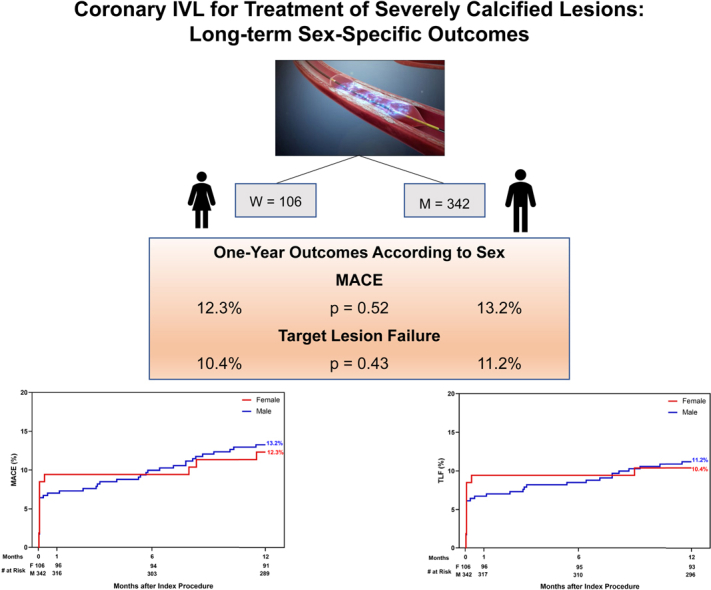

## Introduction

Calcific coronary artery disease is common and presents a challenge in achieving adequate early and late results during percutaneous coronary intervention (PCI). Placement of drug-eluting stents (DES) in calcific lesions that are not optimally prepared or pretreated can lead to stent underexpansion or malapposition, which is associated with higher rates of stent thrombosis, restenosis, and target vessel failure.^1^, ^2^, ^3^ Patients with calcified lesions are common due to an aging population and a high prevalence of diabetes, hypertension, and renal insufficiency.^4^ Interventional treatment of calcified lesions is associated with worse acute and long-term outcomes.^4^, ^5^, ^6^ Prior therapies have focused on the use of noncompliant or specialty (cutting, scoring) balloons, rotational atherectomy, and orbital atherectomy; however, atherectomy is associated with increased periprocedural complications including coronary dissections, perforations, and higher rates of periprocedural myocardial infarction (MI).^7^, ^8^, ^9^ Furthermore, treating calcified lesions in women has been particularly challenging. Female sex is associated with higher mortality, MI, stent thrombosis, and target lesion revascularization after PCI of calcified coronary lesions,^10^ and the use of rotational and orbital atherectomy in women is associated with worse outcomes due to higher procedural complications including severe coronary dissection, cardiac tamponade, and bleeding.^11^^,^^12^

Intravascular lithotripsy (IVL) (Shockwave Medical Inc) is a catheter-based therapy in which lithotripsy emitters enclosed in an integrated contrast-filled balloon generate sonic pressure waves creating a field effect to treat calcium. The pressure waves selectively disrupt and fracture calcium, altering vessel compliance while minimizing injury and maintaining integrity of the fibroelastic component of the vessel wall.^13^ Safety and efficacy of IVL for lesion preparation prior to DES delivery in patients with severely calcific disease has been established in the Disrupt CAD I through IV studies.^13^, ^14^, ^15^, ^16^, ^17^ The short-term sex-specific outcomes of IVL from Disrupt CAD I through IV were recently reported, showing similar safety and efficacy in women when compared with men at 30 days.^18^ In this study, we sought to evaluate the 30-day and 1-year sex-specific outcomes of patients with severely calcified lesions from Disrupt CAD III and IV cohort undergoing lesion preparation with IVL prior to DES implantation.

## Methods

### Study population and objectives

This is a sex-based comparison of 1-year outcomes from a patient-level pooled analysis of the Disrupt CAD III and IV studies. These were prospective, single-arm, multicenter studies evaluating coronary IVL-facilitated stenting for de novo severely calcified coronary artery lesions in patients with stable angina, unstable angina, or silent ischemia. Study designs, detailed inclusion criteria, and outcomes of the Disrupt CAD III and IV studies have been described and published previously.^15^^,^^17^^,^^19^ Both studies were performed in accordance with the institutional review board or ethics committee approval obtained for each study at the participating centers, and all patients provided written informed consent. Shockwave Medical Inc sponsored the studies; investigators had full and open access to the data.

The eligibility criteria and identification of calcified lesions were similar in both studies. Independent angiographic and optical coherence tomography (OCT) core laboratories (Cardiovascular Research Foundation) performed quantitative and qualitative analysis of all images. Severe calcification was defined by OCT demonstrating a calcium angle ≥270 degrees in at least 1 cross-section or by angiographic appearance of radiopacities involving both sides the arterial wall of at least 15 mm in length. IVL followed by second-generation DES placement was also performed consistently in each trial. Clinical follow-up was performed at 1 year for both the Disrupt CAD III and IV studies.

### Study end points

The primary safety end point was freedom from major adverse cardiac events (MACE) at 30 days defined as cardiac death, all MI, and target vessel revascularization. The primary effectiveness end point was procedural success defined with 2 threshold criteria: residual stenosis <50% and residual stenosis ≤30%. Outcomes at 1 year included sex-specific outcomes of MACE, target lesion failure (TLF: composite of cardiac death, target vessel MI, or ischemia-driven target lesion revascularization), and stent thrombosis. Periprocedural MI was defined in both studies as the peak post-PCI creatine kinase myocardial band level >3× the upper limit of normal with or without new pathologic Q-waves. Spontaneous (nonprocedural) MI after discharge was defined according to the Fourth Universal Definition of MI.^20^ All MACE, TLF, and stent thrombosis events were adjudicated by an independent clinical events committee (Cardiovascular Research Foundation).

### Statistical analysis

All primary analyses were performed in the intent-to-treat cohort pooled from the Disrupt CAD III and CAD IV studies. Both parametric and nonparametric summaries of continuous variables are presented. Categorical variables are summarized as counts and percentages.

Factors that may confound the relationship between sex and MACE at 1 year were selected a priori based on historical relatedness to adverse events after calcified lesion PCI and on expert opinion. These factors were: age (per 10 years), baseline body mass index, diabetes, hyperlipidemia, smoking, hypertension, history of stroke or transient ischemic attack, prior MI, lesion location of left anterior descending coronary artery, lesion length (per 10 mm), bifurcation, estimated glomerular filtration rate (<60 mL/min/1.73 m^2^), left ventricular ejection fraction (≥50%), and reference vessel diameter >3.0 mm. Univariate analyses of these potential confounding variables’ relationship with sex and with each outcome variable were completed. Factors with a *P* value of ≤.20 for the associations with sex and with the outcome variable were considered in the multivariable analysis.

The final multivariable Cox proportional hazards models for MACE at 1 year and TLF at 1 year included sex and potential confounders identified through the univariate analyses as well as clinically important factors identified by experts. For both models, the assumption of proportional hazards was tested for each independent variable using a Kolmogorov-type Supremum test, and the assumption was met for all included variables. Sample size limitations prevented the identification of effect modifiers of the relationship between sex and MACE and between sex and TLF.

Assuming the same sample size and sex distribution as this cohort, as well as using the observed rate of MACE in men as a reference, we would have at least 80% power to detect a true hazard ratio (HR) of 1.37 or higher. For TLF, using the observed rate of TLF for this sample as a reference, we would have at least 80% power to detect a true HR of 1.41 or higher.

Bias due to unidentified confounders of the estimated HR for MACE can be quantified using an e-value, which estimates the strength of the true relationships an unidentified confounding factor must have with both the dependent variable and the independent variable of interest.^21^ In this sample, if the true HR of sex for MACE is 1.6, the e-value of an unidentified confounder would need to be 2.7 to result in a HR of 1.0.

Level of statistical significance was defined as *P* < .05. All statistical analyses were carried out using SAS version 9.4 (SAS Institute) and R version 2022.07.2+576.

## Results

**Figure 1:**
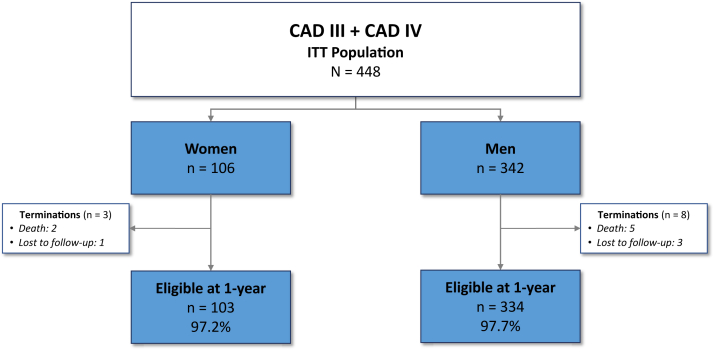
**Patient flow diagram.** ITT, intention-to-treat.

From January 2019 to March 2020, a total of 448 patients including 106 (23.7%) women and 342 (76.3%) men were enrolled at 55 centers in the United States, Europe, and Japan in the Disrupt CAD III and IV studies. Follow-up at 1 year was available in 437/448 (97.5%) of patients (Figure 1). Women were older than men (mean age 73.9 vs 71.0 years) and were less likely to be smokers. Baseline clinical characteristics were otherwise similar between women and men, with similar rates of diabetes, hypertension, prior stroke, prior MI, and renal insufficiency (Table 1). Women had smaller reference vessel diameters (mean 2.8 mm vs 3.1 mm), as well as shorter lesion length (mean 23.6 mm vs 27.1 mm) and total calcified length (mean 44.4 mm vs 49.3 mm) when compared with men. The extent of severe lesion calcium was similar for women and men (Table 1).

**Table 1:** Baseline characteristics according to sex

	Women (n = 106)	Men (n = 342)	*P* value
Age, y	73.9 ± 8.6	71.0 ± 8.5	<.01
Diabetes mellitus	44.3% (47)	40.4% (138)	.47
Hypertension	89.6% (95)	87.7% (300)	.60
Hyperlipidemia	90.6% (96)	88.0% (301)	.47
Prior myocardial infarction	14.2% (15)	19.6% (67)	.21
Prior coronary artery bypass grafting	5.7% (6)	9.4% (32)	.23
Prior stroke or transient ischemic attack	10.4% (11)	9.1% (31)	.69
Smoking/tobacco use	45.3% (48)	59.6% (204)	<.01
Renal insufficiency^a^	8.5% (9)	11.1% (38)	.44
Pacemaker or ICD or CRT-D	4.7% (5)	5.8% (20)	.66
NYHA classification (baseline)			.34
Class I	54.7% (58)	59.9% (205)	–
Class II	45.3% (48)	40.1% (137)	–
Class III	0.0% (0)	0.0% (0)	–
Class IV	0.0% (0)	0.0% (0)	–
CCS angina classification (baseline)			.04
Class I	12.3% (13)	19.9% (68)	–
Class II	40.6% (43)	35.1% (120)	–
Class III	34.9% (37)	26.3% (90)	–
Class IV	0.9% (1)	2.3% (8)	–
Target lesion vessel			.23
Left anterior descending	63.2% (67)	57.9% (198)	–
Right	25.5% (27)	28.1% (96)	–
Left circumflex	11.3% (12)	12.0% (41)	–
Left main	0.0% (0)	2.0% (7)	–
Bypass graft	0.0% (0)	0.0% (0)	–
Reference vessel diameter, mm	2.8 ± 0.4	3.1 ±0.5	<.01
Minimal lumen diameter, mm	1.0 ± 0.4	1.1 ± 0.4	.16
Diameter stenosis, %	64.5 ± 11.5	65.5 ± 10.6	.43
Lesion length, mm	23.6 ± 10.2	27.1 ± 11.8	<.01
Calcification length, mm	44.4 ±16.7	49.3 ± 18.7	.02
Severe calcification^b^	100.0% (106)	100.0% (342)	–
Bifurcation	23.6% (25)	32.7% (112)	.07

Values are % (n) or mean ± SD. *P* values with the Fisher exact test.

CCS, Canadian Cardiovascular Society; ICD/CRT-D, implantable cardiac defibrillator with or without bi-ventricular pacing capability; NYHA, New York Heart Association.

a Estimated glomerular filtration rate <60 mL/min/1.73 m^2^ using the MDRD formula.

b Radiopaque densities noted without cardiac motion generally involving both sides of the arterial wall.

### Procedural characteristics

As shown in Table 2, total procedure time was shorter in women than men. The access site was different between men and women, with women being more likely to have femoral access (42.5% vs 34.5%). Women required fewer lithotripsy catheters and number of pulses compared with men. Women were also treated with fewer stents and shorter total stent length compared with men (30.0 mm vs 33.7 mm).

**Table 2:** Procedural details according to sex

	Women (n = 106)	Men (n = 342)	*P* value
Procedure time, min	52.8 ± 23.7	61.6 ± 29.9	<.01
Contrast volume, mL	158.4 ± 64.3	172.0 ± 71.3	.08
Vascular access			.04
Radial	55.7% (59)	64.9% (222)	–
Femoral	42.5% (45)	34.5% (118)	–
Brachial	0.9% (1)	0.6% (2)	–
Ulnar	0.9% (1)	0.0% (0)	–
Predilatation	45.3% (48)	51.8% (177)	.24
Patients undergoing IVL	96.2% (102)	99.1% (339)	.04
Maximum IVL inflation pressure, atm	6.0 ± 0.2	6.0 ± 0.3	.85
Number of lithotripsy cathters	1.2 ± 0.4	1.3 ± 0.5	<.01
IVL balloon to RVD ratio	1.2 ± 0.1	1.2 ± 0.2	.53
Number of pulses	62.1 ± 33.5	77.5 ± 39.0	<.01
Post-IVL dilatation	14.2% (15)	18.7% (64)	.28
Stent delivery	99.1% (105)	99.4% (340)	.69
Number of stents implanted	1.2 ± 0.4	1.3 ± 0.5	.01
Post-stent dilatation, %	97.2 (103)	98.8 (338)	.23
Total stent length per subject, mm	30.0 ± 12.0	33.7 ± 13.9	.01
Hospital duration, d	1.0 (0.0-1.0)	1.0 (0.0-1.0)	.73

Values are % (n), mean ± SD, or median (range).

IVL, intravascular lithotripsy; RVD, reference vessel diameter.

### Postprocedural and 30-day outcomes

Post-IVL angiographic outcomes including acute gain, minimum lumen diameter, and residual diameter stenosis were similar between women and men (Table 3). Serious angiographic complications, defined as a composite of severe dissection, perforation, abrupt closure, slow flow, or no-reflow, were similar between women and men (2.2% vs 2.6%; *P =* .85). There was no difference in post-IVL severe dissection (type D-F) (3.8% vs 6.7%, *P =* .27) and no difference in residual diameter stenosis between women and men (Table 3). At 30 days, the primary MACE and procedural success end points occurred with similar frequency in men and women (Table 4).

**Table 3:** Angiographic core laboratory-assessed outcomes according to sex

	Women (n = 106)	Men (n = 342)	*P* value
Post-IVL angiographic outcomes^a^			
Acute gain, mm	0.8 ± 0.4	0.8 ± 0.5	.56
Minimal lumen diameter, mm	1.8 ± 0.4	1.9 ± 0.5	.20
Residual diameter stenosis, %	35.2 ± 12.4	38.0 ± 13.6	.09
Post-IVL serious angiographic complications^a^	2.2% (2/90)	2.6% (8/309)	.84
Severe dissection (type D-F)	3.8% (4/106)	6.7% (23/342)	.27
Perforation	0.0% (0/106)	0.0% (0/342)	–
Abrupt closure	0.0% (0/90)	0.0% (0/309)	–
Slow flow	0.0% (0/90)	0.6% (2/309)	.44
No flow	0.0% (0/90)	0.0% (0/309)	–
Final in-segment angiographic outcomes			
Acute gain, mm	1.3 ± 0.4	1.4 ± 0.5	.01
Minimal lumen diameter, mm	2.3 ± 0.4	2.5 ± 0.5	<.01
Residual diameter stenosis, %	17.4 ± 8.1	17.5 ± 8.9	.93
<50%	100.0% (105/105)	99.1% (339/342)	.34
≤30%	93.3% (98/105)	95.9% (328/342)	.28
Final in-stent angiographic outcomes			
Acute gain, mm	1.6 ± 0.4	1.7 ± 0.5	.02
Minimum lumen diameter, mm	2.6 ± 0.4	2.8 ± 0.4	<.01
Residual diameter stenosis, %	10.8 ± 6.8	11.8 ± 6.9	.19
<50%	100.0% (105/105)	100.0% (340/340)	–
≤30%	100.0% (105/105)	99.1% (337/340)	.33
Final – any serious angiographic complications	0.0% (0)	0.6% (2)	.43
Severe dissection (type D-F)	0.0% (0)	0.6% (2)	.43
Perforation	0.0% (0)	0.3% (1)	.58
Abrupt closure	0.0% (0)	0.3% (1)	.58
Persistent slow flow	0.0% (0)	0.0% (0)	–
No flow	0.0% (0)	0.0% (0)	–

Values are % (n) or mean ± SD.

a Post-IVL angiographic data capture was not required per protocol in the Disrupt CAD studies.

**Table 4:** In-hospital and 30-day outcomes according to sex

	Women (n = 106)	Men (n = 342)	*P* value
In-hospital major adverse cardiac events	8.5% (9)	6.4% (22)	.47
Cardiac death	0.0% (0)	0.3% (1)	.58
All myocardial infarction	8.5% (9)	10.2% (35)	.60
Non-Q-wave	8.5% (9)	5.0% (17)	.18
Q-wave	0.0% (0)	1.2% (4)	.26
Target vessel revascularization	0.0% (0)	0.6% (2)	.43
30-day major adverse cardiac events	9.4% (10)	7.0% (24)	.42
Cardiac death	0.9% (1)	0.3% (1)	.38
Myocardial infarction	8.5% (9)	6.7% (23)	.60
Non-Q-wave	8.5% (9)	5.3% (18)	.23
Q-wave	0.9% (1)	1.5% (5)	.68
Target vessel revascularization	0.9% (1)	1.5% (5)	.68
Procedural success			
Residual stenosis <50%	90.6% (96)	93.3% (319)	.35
Residual stenosis ≤30%	90.6% (96)	93.0% (318)	.41
Secondary end points at 30 days			
Target lesion failure	9.4% (10)	6.7% (23)	.35
Cardiac death	0.9% (1)	0.3% (1)	.38
Target vessel myocardial infarction	8.5% (9)	6.7% (23)	.54
Ischemia-driven target lesion failure	0.9% (1)	1.2% (4)	.85
Stent thrombosis (definite or probable)	0.0% (0)	0.9% (3)	.33

Values are % (n).

### One-year outcomes

At 1 year, the rates of MACE were similar for women and men (12.3% vs 13.2%; *P* = .52). At 1 year, the rates of TLF were also similar for women and men (10.4% vs 11.2%; *P* = .43) (Central Illustration and Table 5). After adjustment for major clinical and angiographic covariates, sex was not an independent predictor of MACE at 1 year (HR, 1.24; *P* = .52). Independent predictors for MACE at 1 year included lesion length (HR, 1.45; *P* < .01) and bifurcation lesions (HR, 2.96; *P* < .01) (Table 6). Similarly, after adjustment, sex was not an independent predictor of TLF at 1 year (HR, 1.34; *P* = .43). Independent predictors for TLF at 1 year were also lesion length (HR, 1.44; *P* < .01) and bifurcation lesions (HR, 2.40; *P* < .01) (Table 7).

**Central Illustration:**
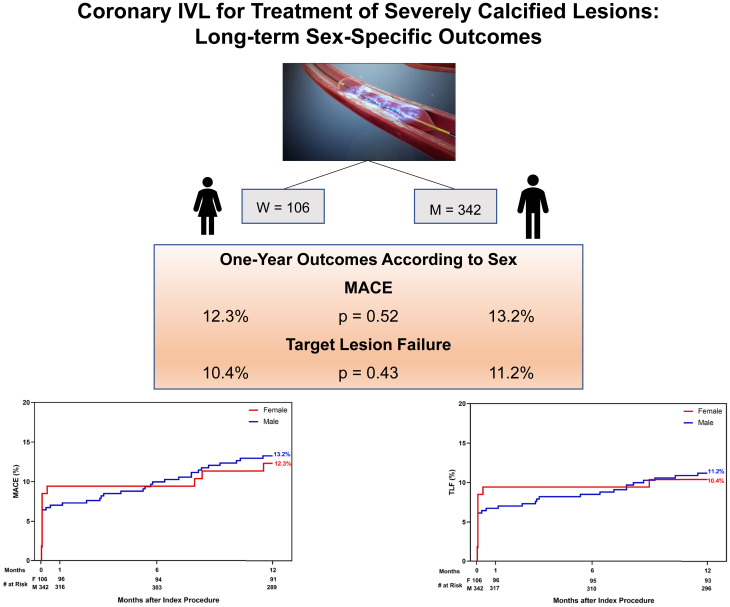
**One-year outcomes of IVL-facilitated stenting of severely calcified coronary lesions according to sex.** IVL, intravascular lithotripsy; MACE, major adverse cardiac event; TLF, target lesion failure.

**Table 5:** One-year outcomes according to sex

	Kaplan–Meier estimates		Unadjusted PH model		Adjusted PH model	
	Women	Men	HR	*P* value	HR	*P* value
Major adverse cardiac events	12.3% (13)	13.2% (45)	0.93 (0.50 - 1.73)	0.82	1.24 (0.64 - 2.40)	0.52
Cardiac death	1	3				
All myocardial infarction	9	35				
Non-Q-wave	9	30				
Q-wave	1	5				
Target vessel revascularization	5	20				
Target lesion failure	10.4% (11)	11.2% (38)	0.94 (0.48 - 1.83)	0.85	1.34 (0.65 - 2.75)	0.43
Cardiac death	1	3				
Target vessel myocardial infarction	9	33				
Ischemia-driven target lesion revascularization	3	14				
Stent thrombosis (definite or probable)	0	3				

Values are Kaplan–Meier estimates, % (n).

HR, hazard ratio; PH, proportional hazards.

**Table 6:** Independent predictors of major adverse cardiac events at 1 year

Independent variable	Hazard ratio	Chi-square	*P* value
Female vs male	1.24	0.41	.52
Lesion length (per 10 mm)	1.45	10.11	<.01
Bifurcation (yes)	2.06	6.77	<.01
Age (per 10 y)	1.33	2.91	.09
Diabetes	1.51	2.17	.14
Prior myocardial infarction	1.57	2.04	.15
Smoker	1.38	1.22	.27
Left ventricular ejection fraction ≥50%	0.76	0.51	.47
Reference vessel diameter >3.0 mm	0.96	0.02	.89

**Table 7:** Independent predictors of target lesion failure at 1 year

Independent variable	Hazard ratio	Chi-square	*P* value
Female vs male	1.34	0.63	.43
Bifurcation (yes)	2.40	8.38	<.01
Lesion length (per 10 mm)	1.44	7.97	<.01
Age (per 10 y)	1.35	2.67	.10
Left ventricular ejection fraction ≥50%	0.59	1.74	.19
Smoker	1.48	1.50	.22
Prior myocardial infarction	1.37	0.77	.38
Diabetes	1.30	0.75	.39
Reference vessel diameter >3.0 mm	0.87	0.21	.65

## Discussion

This pooled analysis from the Disrupt CAD III and IV studies represents the largest 1-year sex-specific outcome following use of IVL to treat severely calcified coronary lesions prior to DES implantation. The major findings are that measures of safety and effectiveness remain favorable and similar in both women and men at 1-year follow-up and that after adjustment for clinical and angiographic covariates, sex was not an independent predictor of MACE or TLF at 1 year.

Our results show that women have smaller vessels when compared with men, but the extent of severe calcification is similar among men and women. Pooled OCT data from patients from Disrupt CAD I-IV echoed that women have smaller minimum lumen area when compared with men, resulting in smaller mean stent area in women. However, calcium morphology and calcium fracture were similar between women and men. Furthermore, there was no difference in stent expansion or strut malapposition between the groups after using IVL. This suggests similar efficacy between men and women at the time of stent implantation.

PCI of calcified lesions has been a longstanding challenge for interventional cardiologists. Despite the use of second-generation DES, both early and late adverse events after PCI of calcified lesions remains high.^22^ Adequate lesion preparation reduces the risk of adverse events, and suboptimal preparation of these lesions can lead to stent underexpansion and malposition, resulting in increased rates of stent thrombosis, restenosis, and target vessel failure.^1^, ^2^, ^3^ Existing strategies of lesion preparation with rotational and orbital atherectomy entail a risk of periprocedural complications, severe dissections, and tamponade, especially in women.^7^, ^8^, ^9^ In a registry of 1622 women undergoing DES implantation, outcomes of women with moderate and severe calcific coronary disease was significantly worse at 3 years compared with those with mild or noncalcified lesions, with an increased risk of death, MI, and target vessel revascularization despite use of second-generation DES^10^; use of rotational atherectomy and orbital atherectomy have not clearly improved outcomes in women compared with men.^11^^,^^12^ This is perhaps explained by smaller vessel size and increased vessel tortuosity in women and more vascular complications (including bleeding) post-PCI in women.^12^ Alternative treatments for women with severely calcified coronary lesions are needed to improve their outcomes.

The short-term outcomes after coronary IVL in women have been previously described.^18^ The outcomes we report at 1-year extend the 30-day results, showing similar safety and efficacy between women and men at the 1-year mark in this high-risk cohort of patients. Although MACE rates exceed 10% in both men and women, this is predominantly driven by MI in both groups. Similarly, TLF is a composite inclusive of MI. Periprocedural MI is defined as peak post-PCI creatine kinase myocardial band level >3× the upper limit of normal with or without new pathologic Q-waves, which is a liberal definition and explains the seemingly high event rates. These rates are similar to what has been seen in other studies including Disrupt CAD III.^16^ Beyond the clinical benefits, IVL is technically simpler to use than rotational and orbital atherectomy, and although no studies exist directly comparing IVL with atherectomy, the event rates up to 1 year have been favorably low with IVL.

### Limitations

Disrupt CAD III and IV are both single-arm studies without a concomitant control population. No studies exist at this point comparing patients receiving IVL with those receiving balloon angioplasty, rotational atherectomy, or orbital atherectomy for pretreatment of calcified coronary lesions, representing a significant evidence gap in need of future investigation. This study is a retrospective analysis of Disrupt CAD III and IV, neither of which were designed to study the difference in men vs women. In addition, even though this is the largest study to date examining the 1-year sex-specific outcomes after IVL, the sample size in this study is modest, with 448 total patients including only 106 (23.7%) women; as such, it is underpowered to detect small differences in outcomes between the sexes. The Disrupt CAD III and IV studies included patients presenting with stable angina, unstable angina, and silent ischemia, and thus, results cannot be generalized to patients presenting with acute coronary syndromes. In addition, these studies excluded patients with lesions that were ostial, bifurcation lesions, unprotected left main disease, in-stent restenosis, >40 mm in length, extremely tortuous, nondilatable, and bypass grafts. Long-term safety and efficacy of IVL beyond 1 year has not yet been studied. Very long-term follow-up (eg, 5 years) is required to characterize lesion stability after IVL treatment of severely calcified lesions. Finally, angiographic follow-up was not performed and thus, whether there are differences in late loss and restenosis after IVL in men and women is unknown.

## Conclusions

In this patient-level pooled analysis from the Disrupt CAD III and IV studies, use of IVL for lesion preparation of severely calcified lesions had similar safety and effectiveness in women and men at 1-year follow-up. The high acute procedural success rate with infrequent complications and similar 1-year clinical outcomes after IVL prior to DES implantation in severely calcified lesions in women and men is overall favorable, suggesting that use of IVL in this high-risk cohort is safe.
